# AI-driven insights into protein misfolding and innate immunity in neurodegenerative diseases

**DOI:** 10.3389/fimmu.2026.1814357

**Published:** 2026-05-12

**Authors:** Hui Xin Deng, Jing Ling Cao, Yao Wu, Si Jin Jiang, Qian Qian Fang, Bi Yue Zhu, Yong Jian Jiang

**Affiliations:** Department of Pharmacy Children‘s Hospital of Chongqing Medical University, National Clinical Research Center for Children and Adolescents’Health and Diseases, Ministry of Education Key Laboratory of Child Development and Disorders, International Science and Technology Cooperation Base of Child Development and Critical Disorders, Chongqing Key Laboratory of Child, Neurodevelopment and Cognitive Disorders, Intelligent Application of Big Data in Pediatrics Engineering Research Center of Chongqing Education Commission of China, Chongqing, China

**Keywords:** artificial intelligence, innate immune, misfolded proteins, neurodegenerative disease, neuroinflammation

## Abstract

Neurodegenerative diseases encompass a diverse group of disorders ranging from adult-onset conditions such as Alzheimer’s and Parkinson’s disease to pediatric forms including neuronal ceroid lipofuscinoses (NCLs), Niemann-Pick type C (NPC), and infantile neuroaxonal dystrophy (INAD), all of which are characterized by protein misfolding and chronic neuroinflammation. During their occurrence and development, the innate immune system, especially the immune responses mediated by microglia in the central nervous system, plays a crucial regulatory role. Increasing evidence indicates that misfolded and abnormally aggregated proteins, such as β-amyloid (Aβ), Tau, α-synuclein, and TDP-43, are not only neurotoxic factors but can also act as damage-associated molecular patterns (DAMPs) recognized by innate immune receptors, thereby triggering persistent neuroinflammatory responses. However, traditional experimental and computational methods still have significant limitations in systematically analyzing the “protein misfolding–innate immune activation” mechanism. In recent years, artificial intelligence has made breakthrough progress in protein structure prediction, multi-conformation modeling, and integration of multi-omics data, providing a new research paradigm for revealing the intrinsic relationship between protein misfolding and innate immunity across the spectrum of neurodegenerative diseases. This article systematically reviews the latest applications of artificial intelligence in predicting the conformational characteristics of misfolded proteins, simulating the protein aggregation process, revealing the mechanism of innate immune perception, and reconstructing the regulatory network of neuroinflammation. It focuses on discussing the significance of deep learning models such as AlphaFold, I-TASSER, RoseTTAFold, Phyre2, and ESMFold in the field of protein structure prediction, as well as the related research on multi-modal AI technology in revealing the complex molecular mechanisms behind neurodegenerative diseases, such as combining AI with mathematical models to simulate the spread of misfolded proteins and further exploring the association with disease progression. The review also highlights the potential of AI to address the diagnostic challenges unique to pediatric neurodegenerative disorders, which, despite their rarity, collectively impose devastating lifelong burdens. In summary, AI tools not only deepen our understanding of the molecular mechanisms underlying both adult and childhood neurodegenerative diseases but also open up new avenues for developing innovative diagnostic tools and treatment methods.

## 1 Introduction

Neurodegenerative diseases, including Alzheimer’s disease, Parkinson’s disease, amyotrophic lateral sclerosis (ALS), Huntington’s disease, and pediatric-onset disorders such as neuronal ceroid lipofuscinoses (NCLs), Niemann-Pick type C (NPC), and infantile neuroaxonal dystrophy (INAD) (1–4), are a group of disorders characterized by progressive neuronal loss and mainly manifested as cognitive or physical dysfunction. In recent years, with the increase in the global aging population, the prevalence of neurodegenerative diseases has significantly increased (5–7), particularly Alzheimer’s disease and Parkinson’s disease, which have been the focus of extensive mechanistic studies (8). While the global rise in aging populations has driven increasing prevalence of adult forms, childhood neurodegenerative diseases, though individually rare, collectively impose devastating lifelong burdens and share core molecular features with their adult counterparts, including protein misfolding and chronic neuroinflammation.

**Figure 1:**
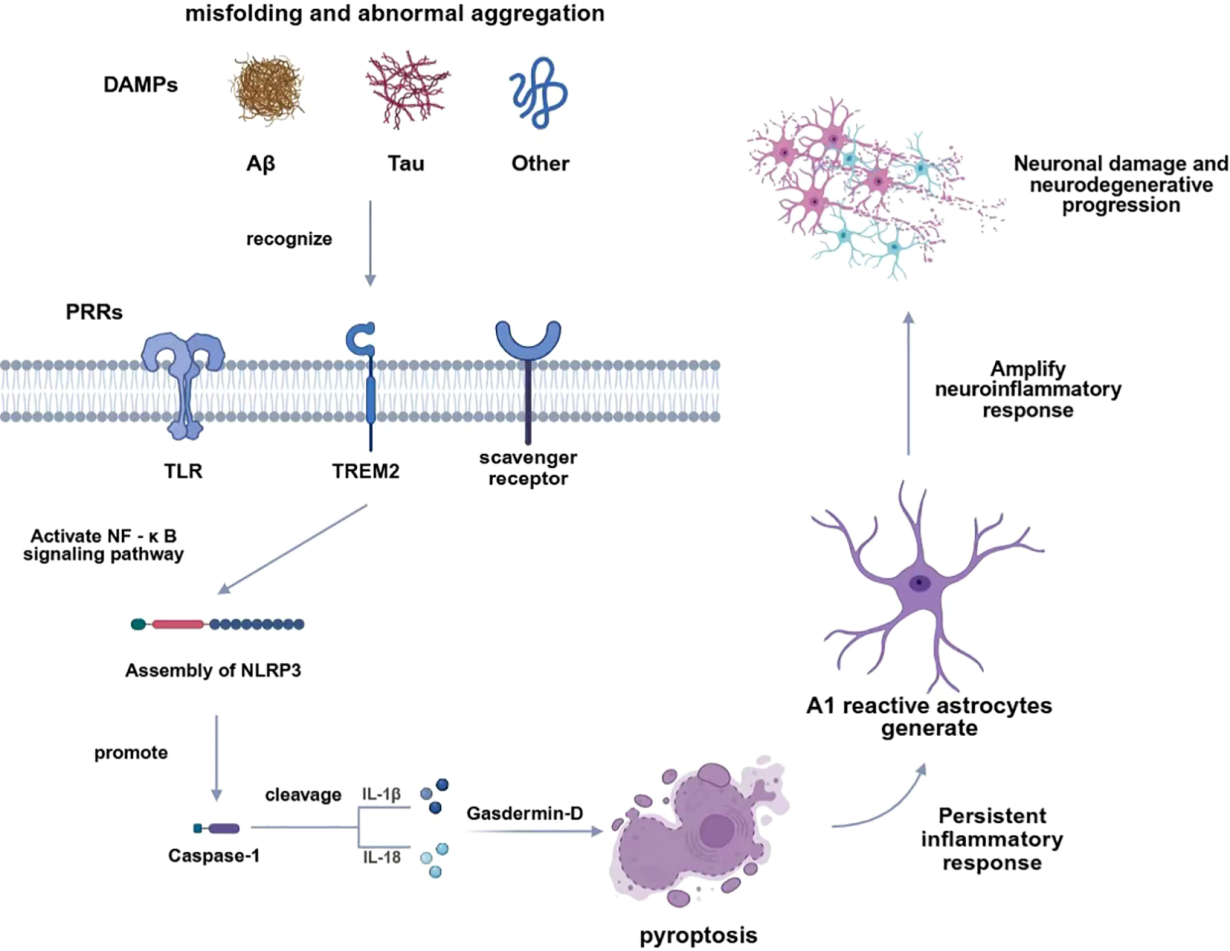
Innate immune mechanisms of misfolded proteins in neurodegenerative diseases. Schematic illustration of the regulatory axis between misfolded proteins (Aβ, Tau, α-synuclein, etc.) and CNS innate immune responses. Misfolded proteins act as DAMPs to be recognized by PRRs on microglia and astrocytes, inducing microglial M1/M2 polarization and astrocytic A1/A2 phenotypic shift, which further triggers NLRP3 inflammasome activation and pro-/anti-inflammatory cytokine secretion. The bidirectional effects of innate immune activation—mild activation for misfolded protein clearance and excessive activation for neuronal damage—are also depicted to reveal the cascade link between protein misfolding and chronic neuroinflammation.

Misfolded proteins, such as tau (9, 10), α-synuclein (11), TDP-43 (12, 13), and amyloid-β (14, 15) proteins, play a crucial role in the pathogenesis of neurodegenerative diseases. The abnormal aggregation of these proteins to form amyloid fibrils defines most human neurodegenerative diseases. Abnormal folding and aggregation of tau protein and amyloid-β proteins are typically associated with Alzheimer’s disease (14, 16). α-synuclein is more common in Parkinson’s disease (17), Parkinson’s disease with dementia, dementia with Lewy bodies, and multiple system atrophy (MSA) (18). Abnormal assembly of TDP-43 is a characteristic of the vast majority of ALS cases and approximately half of frontotemporal dementia cases (12). Neuroinflammation is an inflammatory response within the brain or spinal cord, coordinated by inflammatory mediators (such as cytokines, chemokines, etc.) produced by central nervous system inflammatory cells (such as microglia, oligodendrocytes, etc.) (19), including immune cell infiltration, microglial activation, and pro-inflammatory cytokine release. Various studies have shown that long-term or maladaptive neuroinflammation plays a central role in various neurological disorders, including neurodegenerative diseases (20, 21), mental disorders (22, 23), pain syndromes (24), stroke (25, 26), and traumatic brain injury (27, 28).In addition to genetic factors and environmental stress, dietary components may also participate in the regulation of protein aggregation. A recent study has shown that the widely used food additive monosodium glutamate (MSG) can induce protein aggregation through a nucleation-dependent aggregation mechanism, accompanied by significant alterations in secondary structure, providing new insights into the link between environmental factors and neurodegenerative diseases (29).Protein misfolding and neuroinflammation constitute the common pathological manifestations of neurodegenerative diseases (**Figure 1**).

Schematic illustration of the regulatory axis between misfolded proteins (Aβ, Tau, α-synuclein, etc.) and CNS innate immune responses. Misfolded proteins act as DAMPs to be recognized by PRRs on microglia and astrocytes, inducing microglial M1/M2 polarization and astrocytic A1/A2 phenotypic shift, which further triggers NLRP3 inflammasome activation and pro-/anti-inflammatory cytokine secretion. The bidirectional effects of innate immune activation—mild activation for misfolded protein clearance and excessive activation for neuronal damage—are also depicted to reveal the cascade link between protein misfolding and chronic neuroinflammation.

The innate immune response plays a crucial role in neurodegenerative diseases. In neurodegenerative disorders, cells involved in the innate immune response include not only microglia and astrocytes, but also macrophages, natural killer cells, and mast cells, among others (1, 30). Microglia are common innate immune cells in the central nervous system. Depending on the expression of chemokines and cytokines in the body, their activation can be classified as classical (M1) or alternative (M2) (1). Microglia can secrete pro-inflammatory and anti-inflammatory factors, which may be beneficial or harmful in neurodegenerative diseases. Astrocytes have been reported to produce pro-inflammatory mediators (A1) and immunoregulatory mediators (A2) (31), exerting neuroinflammatory and neuroprotective effects respectively. In summary, the innate immune response occupies an irreplaceable core position in the initiation and progression of neurodegenerative diseases.

The traditional research methods for neurodegenerative diseases mainly include neuroimaging, cerebrospinal fluid biomarker analysis, and clinical evaluation, etc (32). Although these methods are indispensable, they have significant limitations in the early diagnosis, prediction, and precise treatment of the diseases. Although proteomics research provides a platform for mapping protein networks and discovering therapeutic targets, its complexity and the difficulty of data analysis remain challenges (33, 34). In this context, artificial intelligence has emerged as a transformative tool, driving progress across multiple dimensions. It not only significantly enhances the efficiency of biomarker discovery, improves diagnostic accuracy, and accelerates therapeutic development but also optimizes clinical trial design and enables more efficient utilization of multi-omics and neuroimaging data. Collectively, AI is profoundly advancing our understanding of disease mechanisms and continuously driving innovation in treatment strategies (35, 36).

## 2 Molecular mechanisms of protein misfolding and innate immune sensing in neurodegeneration

### 2.1 Protein misfolding in neurodegeneration

#### 2.1.1 The conformational diversity of misfolded proteins

One of the central molecular hallmarks of neurodegenerative diseases is the abnormal misfolding of proteins into structurally heterogeneous and aggregation-prone conformations. Beyond their direct neurotoxic effects, misfolded proteins exhibit pronounced conformational diversity, strain specificity, and propagation properties across different disease contexts. Accumulating evidence indicates that distinct misfolded protein species can be sensed by the innate immune system and function as damage-associated molecular patterns (DAMPs), thereby activating microglia and astrocytes and driving chronic neuroinflammatory responses. Different protein conformations not only contribute to the complexity of neurodegenerative diseases, but also provide potential targets for therapeutic intervention.

The kinetic process of protein aggregation is influenced by multiple factors, including molecular chaperones, polyanions, and others. Studies have shown that heparin can significantly accelerate protein aggregation via a downhill polymerization mechanism, suggesting that exogenous molecules play important roles in the regulation of protein aggregation (37).

The structural basis of protein aggregation has garnered widespread attention in recent years. A variety of structural biology methods have been applied to elucidate the conformational changes during protein aggregation, revealing the intrinsic link between aggregation propensity and local structural features of proteins (38–40). Inhibition strategies for protein aggregation represent an important direction in the treatment of neurodegenerative diseases. The natural osmolyte trehalose has been shown to stabilize protein conformation through the formation of a hydrogen bond network, thereby inhibiting the fibrillar aggregation of α-lactalbumin in a concentration-dependent manner, providing a rationale for the development of small molecule-based protein aggregation inhibitors (41).

##### 2.1.1.1 Amyloid-β

**Figure 2:**
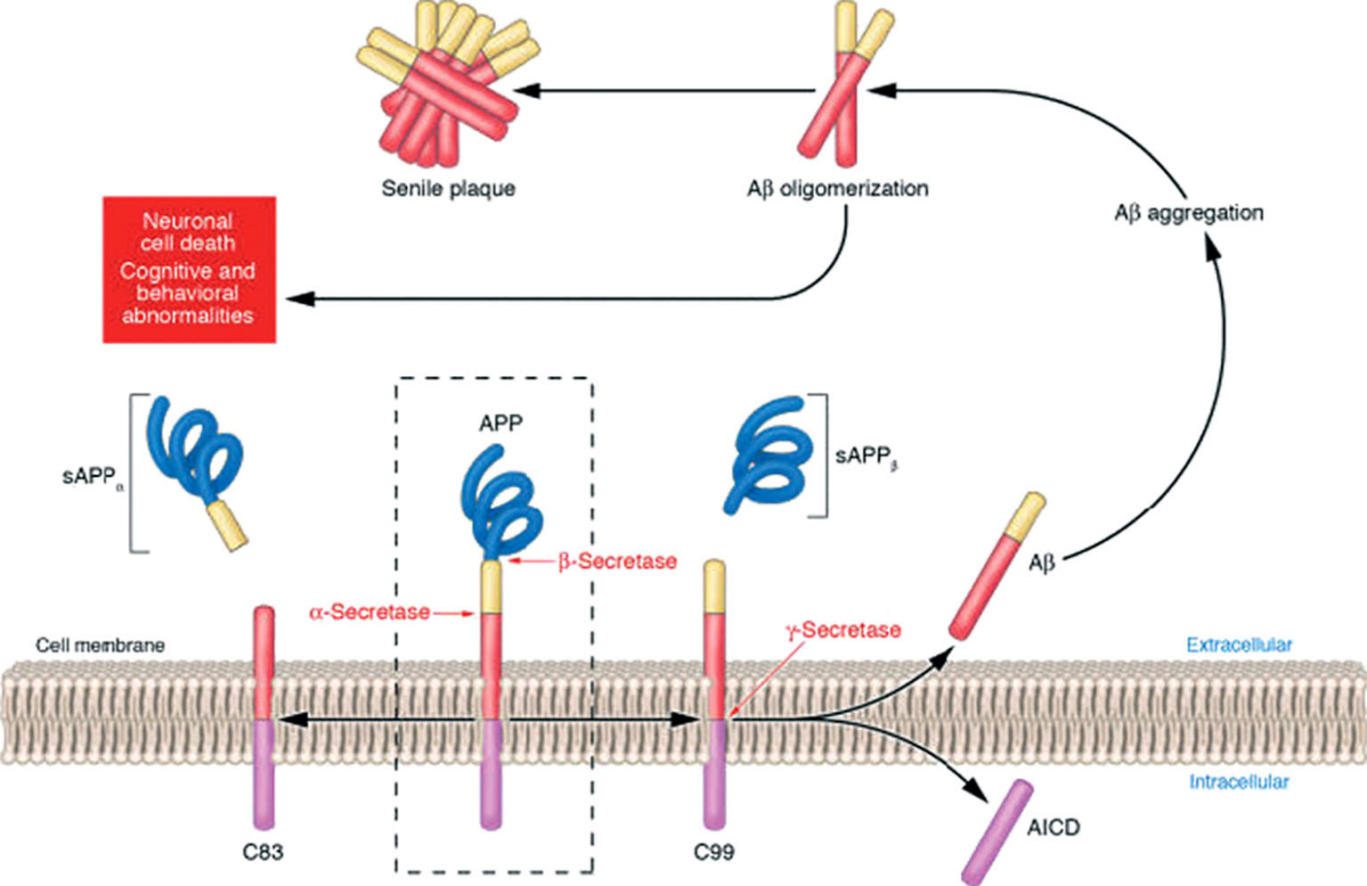
APP is processed by two competing pathways: the α-secretase pathway produces sAPPα and C83, while the β-secretase pathway generates sAPPβ and C99, with some cleavage also producing C89; all C-terminal fragments are then cleaved by γ-secretase to release AICD and Aβ peptides, which aggregate into oligomers that are the most potent neurotoxins, while end-stage senile plaques are relatively inert (51).

Aβ is a small peptide produced by the hydrolysis of amyloid precursor protein (APP). Its polymer forms a pathological lesion known as “amyloid plaques”. The intracellular cleavage of the β-carboxyl-terminal fragment (βCTF) of amyloid precursor protein (APP) results in the production of two proteins, Aβ_40_ and Aβ_42_ (42). These two types are the most common in Alzheimer’s disease (**Figure 2**). In addition, some other types such as Aβ38, Aβ43, Aβ45, Aβ46, Aβ48, Aβ49, and some shorter cleavage products have also been reported (42–44). The post-translational modifications of Aβ protein, including oxidation, phosphorylation, nitration, racemization, isomerization, glutamate pyroglutamylation and glycosylation (45), can give Aβ protein different properties and affect the formation of its oligomers. The oxidation of Met35 alters the characteristic morphology of Aβ fibrils, preventing the formation of protofibrils and inhibiting the formation of oligomers (46, 47). The phosphorylation of serine residue 8 of Aβ has long been proven to increase the formation of Aβ oligomers (48, 49). Nitration of Aβ can initiate plaque formation in APP/PS1 mice, indicating that it plays a core role in the early stage of AD (50). Furthermore, Aβ also forms two types of assemblies. The type 1 oligomers appear in the early stage of AD, while the type 2 oligomers only emerge after the formation of the plaques (43).

##### 2.1.1.2 Tau

Tau protein is the most abundant protein in the neural microtubule system. In the human brain, tau protein is divided into six subtypes, including 0N3R, 1N3R, 2N3R, 0N4R, 1N4R, and 2N4R (43). Similar to Aβ, tau protein also undergoes several types of post-translational modifications, including arginine mono-methylation, lysine acetylation, lysine mono-methylation, lysine di-methylation, lysine ubiquitination, serine O-GlcNAc modification, and serine/threonine/tyrosine phosphorylation (52). The current main focus of attention is the abnormal phosphorylation of tau protein. The abnormal phosphorylation of tau protein is regarded as a pathological feature of Alzheimer’s disease and other tau-related disorders. However, the phosphorylation level of tau protein alone is not sufficient to directly define tau diseases (53). Focusing on the connections between different PTMs may potentially help in identifying new therapeutic targets. In different diseases, the aggregation of tau protein may form different conformations, known as tau strains, such as AD-tau(Alzheimer’s disease), PSP-tau(Progressive supranuclear palsy) and CBD-tau(Corticobasal degeneration) (54). Different tau strains determine the seeding ability of tau aggregation and the specificity of cell types.

##### 2.1.1.3 α-synuclein

α-synuclein is a small molecule protein consisting of 140 amino acids. It is mainly expressed in neurons of the central nervous system, with a particular concentration at the presynaptic terminals (55). α-synuclein also has two isoenzymes, namely β-synuclein and γ-synuclein, and they both belong to the synuclein protein family (56). Similar to tau protein, α-synuclein also has different strains in various diseases, such as PD-strains (Parkinson’s disease), DLB-strains (Lewy body dementia), and MSA-strains (multiple system atrophy). These strains show significant differences in their pathological manifestations (57). Multiple studies have confirmed that MSA α-syn aggregates are different from those in PD and DLB. The Cryo-Em results of MSA show that it has two types of α-syn filaments, while PD and DLB only have one (58, 59). Such differences explain the variations in pathological manifestations.

##### 2.1.1.4 TDP-43

TDP-43 is an RNA-binding protein. Initially, it was discovered to be capable of binding to the TAR DNA in the long terminal repeat (LTR) region of human immunodeficiency virus type 1 (HIV-1) (60). Later, it was confirmed to be a key component of insoluble and ubiquitin-coated inclusions in the brains of patients with amyotrophic lateral sclerosis (ALS) and frontotemporal lobar degeneration (FTLD or FTLD-TDP) (12, 13). The TDP-43 protein contains 414 amino acid residues and is composed of four domains: an N-terminal domain(NTD), two RNA recognition domains and a C-terminal low complexity domain (LCD) (61, 62). NTD can form dimers through a head-to-tail arrangement. Moreover, studies using cryo-electron microscopy and nuclear magnetic resonance (NMR) have confirmed that NTD can also transform into a monomeric alternative folding state, with a lower tendency to polymerize (62, 63). This indicates that it inherently possesses an internal conformational plasticity. Furthermore, LCD has demonstrated multiple structural and biochemical properties in promoting the formation of liquid droplets or inducing fibrillar aggregates (64).

#### 2.1.2 Misfolded proteins as damage-associated molecular patterns

Misfolded proteins can play a crucial role as damage-associated molecular patterns in the innate immune process. The most common one among them is Aβ. In Alzheimer’s disease, microglia can bind to soluble amyloid β (Aβ) oligomers and Aβ fibrils through class A scavenger receptor A1, CD36, CD14, α6β1 integrin, CD47 and toll-like receptors (TLR2, TLR4, TLR6 and TLR9). After Aβ binds to CD36, TLR4 and TLR6, it can activate microglia, generating pro-inflammatory cytokines and chemokines (65). The Aβ in microglia can also trigger the activation of NLRP1 and NLRP3 inflammasomes through different mechanisms (66, 67). In addition, hyperphosphorylated microtubule-binding protein tau(p-tau) and α-synuclein can also act as DAMPs to activate the innate immune process in the context of neurodegenerative diseases (68).

### 2.2 Innate immune sensing of misfolded proteins in the CNS

#### 2.2.1 The innate immune functions of microglia and astrocytes

Within the central nervous system, misfolded and aberrantly aggregated proteins are not only direct neurotoxic agents but also potent endogenous triggers of innate immune activation. Resident innate immune cells, particularly microglia and astrocytes, sense these abnormal protein species as damage-associated molecular patterns (DAMPs) through pattern recognition receptors (PRRs), thereby initiating inflammatory signaling cascades. Among these pathways, inflammasomes—most notably the NLRP3 inflammasome—serve as critical hubs for integrating innate immune signals and amplifying neuroinflammatory responses. In this section, we provide an overview of the innate immune functions of major glial cell populations, outline the mechanisms by which misfolded proteins are recognized in the CNS, and highlight the pivotal role of inflammasome-driven signaling in shaping neuroinflammation during neurodegenerative disease progression.

##### 2.2.1.1 Microglia

**Figure 3:**
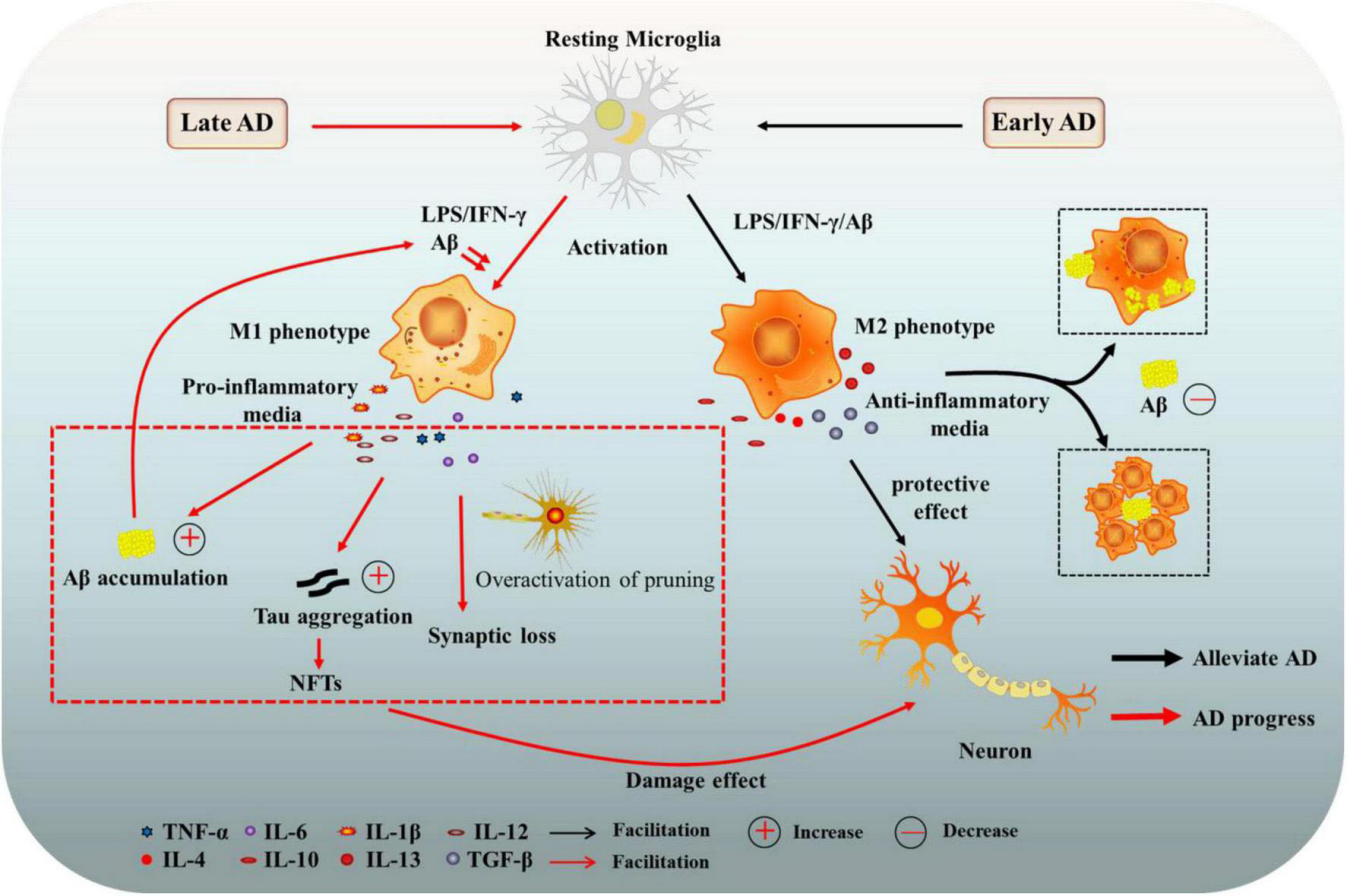
The role of microglia in Alzheimer’s disease (AD). Microglia can have beneficial or harmful effects on AD. In the early stage of AD, the activation state of microglia is mainly a protective phenotype (M2), secreting anti-inflammatory mediators and neurotrophic factors, and clearing or isolating Aβ and Tau proteins, contributing to the protection of neurons. In late AD, microglia tend to be activated as a harmful phenotype (M1), enhancing the release of pro-inflammatory factors, which damage neurons and promote the progression of AD. AD, Alzheimer’s disease; Aβ, amyloid beta-protein; NFTs, neurofibrillary tangles; LPS, lipopolysaccharide; TGF-β, transforming growth factor-β; IFN-γ, interferon γ; TNF-α, tumor necrosis factor-α; IL-6, interleukin-6 (86).

Microglia are widely distributed throughout the central nervous system and are the main innate immune cells (69, 70). When neurons are damaged, microglia will migrate to the affected area, exerting their phagocytic ability to remove debris or eliminate pathogens and adopting a pseudopod-like shape (71, 72). However, new research has found that microglia do not enter a dormant or quiescent state in the so-called “resting” condition. This is different from the traditional “two-state paradigm” (73). *In vivo* studies have shown that branched microglial cells exhibit strong dynamics, with their protrusions constantly moving to monitor the structural regions of the central nervous system. Microglial morphological diversity is governed by several physiological factors, including regional distribution, age, and biological sex (74, 75). Microglia have diverse functions (**Figure 3**). In a healthy organism, microglia play significant roles in synaptic plasticity, neurotrophic support, myelin remodeling, and the maintenance of the internal stability of the central nervous system (76–78).

In the early stage of the disease, microglia cells recognize and engulf Aβ protein by expressing a series of receptors such as TREM2 (79, 80). They form dense nuclear plaques within the cells to prevent the release of toxic Aβ substances (81, 82). Furthermore, microglia cells will also gather at the affected area, forming a barrier to restrict the further spread of the damage (83). When the pathological stimulus persists, microglia enter an over-activated state, at which point their function becomes harmful. The surface of microglia can express various cell receptors, such as toll-like receptors (TLRs), nuclear oligomerization domain-like receptors (NLRs), and viral receptors, which can recognize PAMPs and DAMPs (1, 84). After being stimulated by corresponding factors, microglia can produce pro-inflammatory cytokines such as tumor necrosis factor (TNF)-α, interleukin (IL)-1β, IL-16, and chemokines such as C-C motif chemokine ligand 2 (CCL2) and IL-18 (85). These substances can cause persistent neuroinflammation, serving as the core engine driving the progression of the disease.

##### 2.2.1.2 Astrocytes

Astrocytes are the most common type of cells in the central nervous system and also play a crucial role in the innate immune process (87). As mentioned earlier, astrocytes can transform into two forms: A1 (neurotoxic) and A2 (neuroprotective) (31). The formation of these two different types of cells depends on the induction of microglia (88, 89). The A1 astrocytes upregulate various inflammatory markers, such as ROS, IL-1β, and TNF-α (90). The A1-reactive astrocytes exhibit many functional impairments compared to most other astrocytes, such as a reduced ability to induce synaptic formation and function, a loss of the ability to phagocytose synapses, and a loss of the ability to promote neuronal survival and growth. It is reported that A1 reactive astrocytes secrete a neurotoxin that induces apoptosis of neurons and oligodendrocytes (31). As depicted in the figure, A1 cells can establish and amplify the local inflammatory microenvironment by releasing chemokines (Mechanism A), which mediate the chemotaxis and recruitment of immune cells (87). In A2 astrocytes, what is upregulated are protective mediators, including protein prokineticin-2 (PK2), chitin-like 3, Frizzled class receptor, arginase 1, NF-E2-related factor 2 (Nrf2), pentraxin 3, sphingosine kinase 1, and transmembrane 4 L6 family member 1 (31, 91). Therefore, the functions of A2 reactive astrocytes mainly focus on promoting synaptic repair, growth, and neuronal survival (92). Concurrently, A2 cells may regulate adaptive immune responses and coordinate tissue repair through mechanisms such as antigen presentation (Mechanism C), for instance, by forming MHC-II/TCR complexes to activate specific T cell subsets (87).

#### 2.2.2 Pattern recognition receptors and misfolded proteins

PRRs are a group of receptors that are involved in recognizing PAMPs or DAMPs, including mitochondrial DNA (mtDNA), ATP, Cyclophilin A, HMGB1, β-defensins, histones, calreticulin, syndecans, glypicans, HSPs, and S100 proteins (93–95). PRRs are expressed in cells involved in innate immunity, such as microglia, astrocytes, and oligodendrocytes (96). Furthermore, as mentioned earlier in the text, misfolded proteins can also act as DAMPs and be recognized by PRRs in the context of neurodegenerative diseases. Among the neurodegenerative diseases, the PRR types that have been most thoroughly studied include TLRs, CDSs, NLRs and ALRs, which together form the inflammasome (68). Inflammasomes are cytoplasmic polymeric complexes that can produce important innate immune mediators, such as IL-1β, IL-18 (97), caspase-1, and Gasdermin D (GSDMD) (98). Multiple studies have shown that the NLRP3 inflammasome plays a crucial pathogenic role in various neurodegenerative diseases (98, 99), Recent advances have further elucidated the context-specific roles of inflammasome activation in Alzheimer’s and Parkinson’s disease (100).

#### 2.2.3 The influence of inflammasomes on neuroinflammation

Multiple signals in neurodegenerative diseases can abnormally activate NLRP3, thereby driving the progression of the disease. The successful activation of the NLRP3 inflammasome requires two signals. The initiating signals provided by NF-κB activation stimuli will promote the transcriptional expression of NLRP3 and pre-IL-1β (101). The activation signals are provided by various NLRP3 activators, including PAMPs, aggregated and misfolded proteins, ATP, and crystalline substances (102–106). After NLRP3 is assembled and activated, it triggers the maturation of IL-1β and IL-18 (107). IL-1β can further activate the surrounding microglia and astrocytes, causing them to produce more pro-inflammatory factors (such as TNF-α and IL-6) (107). IL-18 not only induces the production of pro-inflammatory factors but also enhances the expression of Fas ligand in glial cells, thereby exacerbating Fas-mediated neuronal cell death in neuroinflammation (107). Furthermore, inflammasomes can also trigger pyroptosis. The activation of inflammasomes leads to the activation of Caspase-1 and Gasdermin-D, and allows the secretion of pro-inflammatory IL-1 family cytokines (108). Gasdermin-D oligomerizes at the plasma membrane, resulting in pore formation, release of cell contents, and ultimately cell lysis (109).

## 3 AI-driven approaches for studying protein misfolding and immune responses

### 3.1 AI approaches for studying protein misfolding

Protein misfolding and aberrant aggregation represent central molecular events in the onset and progression of neurodegenerative diseases, yet their structural characterization has long been constrained by the limited availability of experimental structures.The structural characterization of misfolded proteins has historically relied on experimental techniques, whose limitations have been extensively reviewed (110). Recent advances in artificial intelligence–driven protein structure prediction have fundamentally reshaped this landscape, spanning approaches from classical template-based assembly frameworks to end-to-end deep learning models and protein language models. These methodologies offer complementary strengths in resolving stable folding cores, modeling multidomain architectures, identifying intrinsically disordered regions, and assessing the structural impact of disease-associated mutations. Together, they constitute a rapidly evolving computational toolkit for dissecting the structural basis of protein misfolding. In this section, we summarize representative AI-based structure prediction models, highlighting their underlying principles, applications to misfolding-related proteins, and inherent limitations in the context of neurodegenerative disease research.

#### 3.1.1 AlphaFold series

**Figure 4:**
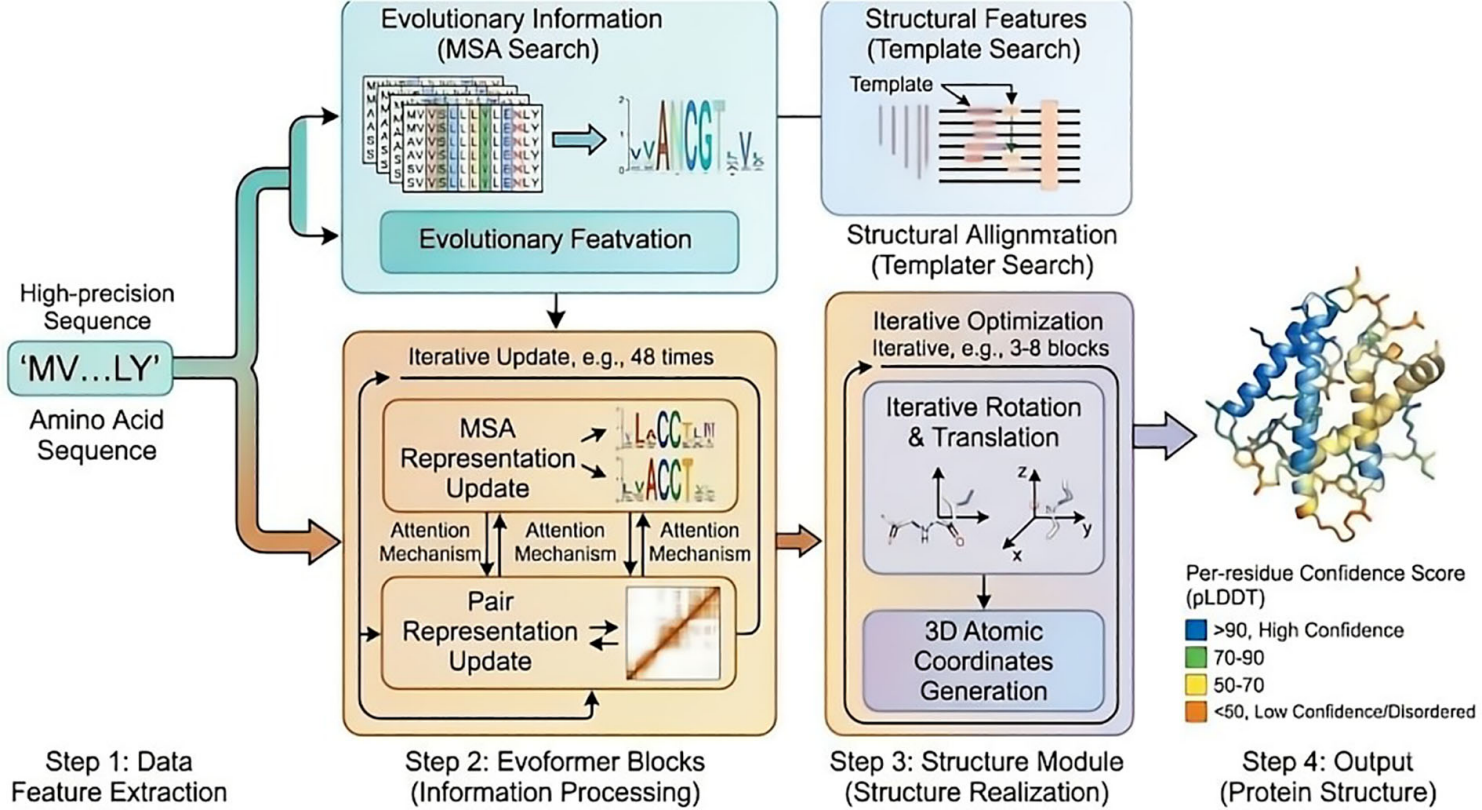
Workflow of AlphaFold2 for *de novo* protein three-dimensional structure prediction. Workflow of AlphaFold2 for de novo protein three-dimensional structure prediction. Schematic of the AlphaFold2 pipeline for high-precision protein structure prediction, starting with amino acid sequence input. Evolutionary information is obtained via MSA and structural features via template alignment, followed by Evoformer module-mediated integration of sequence-spatial features to capture inter-residue co-evolution and long-range interactions. The structure module performs iterative optimization to generate atomic-level 3D models, with final output of protein conformations (main/side chains) and per-residue confidence scores (pLDDT) for structural reliability evaluation.

The AlphaFold series of models represents a milestone breakthrough in the field of protein structure prediction, with its core innovation lying in the end−to−end integration of deep learning with evolutionary information from protein sequences and spatial geometric constraints (111–113). By learning from large−scale multiple sequence alignment (MSA) data, the model systematically captures co−evolutionary relationships between residues (114–116). Combined with attention mechanisms and geometric reasoning modules, it accurately models long−range interactions in proteins, thereby achieving high−precision prediction of three−dimensional protein structures without explicitly introducing traditional physical energy functions (114–118). Particularly in AlphaFold2, the introduced Evoformer architecture and structure module enable iterative updates between sequence representations and spatial representations, endowing the model with the ability to directly infer spatial conformation from sequences (**Figure 4**). Its prediction accuracy in multiple international structure prediction assessments approaches or even reaches experimental resolution levels (119, 120). Following AlphaFold2, its latest iteration, AlphaFold 3, was released in 2024, achieving significant breakthroughs in architecture and functionality (121). Compared to AlphaFold2, AlphaFold 3 employs a simplified Pairformer module in place of the original Evoformer and introduces a diffusion module to directly generate three−dimensional atomic coordinates, substantially improving the prediction of protein–small molecule interactions, protein-nucleic acid interactions, and post−translational modification sites (122). This advance holds particular value for investigating the “protein misfolding-innate immune sensing” axis in neurodegenerative diseases (123). AlphaFold 3 provides a more direct and accurate computational tool for structural modeling of such complex interaction complexes, enabling the prediction of a broader range of biomolecular structures and their interactions through more comprehensive integration of sequence, structural, and chemical contexts (124, 125).

Schematic of the AlphaFold2 pipeline for high-precision protein structure prediction, starting with amino acid sequence input. Evolutionary information is obtained via MSA and structural features via template alignment, followed by Evoformer module-mediated integration of sequence-spatial features to capture inter-residue co-evolution and long-range interactions. The structure module performs iterative optimization to generate atomic-level 3D models, with final output of protein conformations (main/side chains) and per-residue confidence scores (pLDDT) for structural reliability evaluation.

This technological breakthrough significantly lowers the barrier to obtaining protein structures, enabling systematic resolution of a large number of human proteins that previously lacked experimental structural information, including many key proteins and their variants closely associated with neurodegenerative diseases. For example, AlphaFold−predicted structural models of proteins such as Tau (126, 127) and TDP−43 (128, 129) provide important references for understanding their domain composition, potential folding cores, and the impact of mutations on local structural stability. Moreover, the per−residue confidence score (pLDDT) output by AlphaFold can, to some extent, be used to identify flexible regions and potentially disordered segments in proteins (130–133), which is of particular value for analyzing the intrinsically disordered regions (IDRs) widely present in proteins related to neurodegenerative diseases (134, 135).

In the context of neurodegenerative disease research, AlphaFold is not only significant as a structural prediction tool itself, but also provides a critical “structural starting point” for subsequent studies. Its predicted structures can be employed in molecular dynamics simulations, modeling of protein aggregation processes, and protein− receptor interaction analyses, thereby assisting in exploring how misfolded proteins acquire aberrant conformations and participate in neurotoxicity and immune activation processes. However, it is important to note that AlphaFold primarily predicts a single stable conformation and cannot directly reflect the conformational diversity and dynamic transition characteristics exhibited by misfolded proteins under disease conditions (136–140). This also suggests that in neurodegenerative disease research, it is more suitable as a foundational module within a multiscale computational analysis framework rather than a complete solution.

It should be noted that AlphaFold and its subsequent versions were originally designed to predict native state conformations of proteins (116). However, a central pathological feature of neurodegenerative diseases lies precisely in the deviation of proteins from their native states into kinetic traps of non-native conformations, leading to misfolding and aggregation (141, 142). Therefore, while AlphaFold serves as a powerful generative prior, its predictions in the context of non-native states necessitate rigorous cross-validation with experimental data. Notably, the pLDDT scores output by AlphaFold provide important clues for understanding the propensity for protein misfolding (131). For proteins containing extensive intrinsically disordered regions (IDRs), such as Tau and TDP-43 (143, 144), AlphaFold often yields low-confidence predictions. Rather than representing a “failure”, this accurately reflects the inherent property of these regions to lack stable folding under physiological conditions and to exhibit high conformational heterogeneity (145). Such “conformational frustration” constitutes the molecular basis for driving aberrant phase transitions and pathological aggregation. Therefore, AlphaFold can be regarded as a generative prior, providing a structural starting point, rather than a static endpoint, for exploring the non-native energy landscapes that govern transitions from native to misfolded states (145). Recent studies indicate that AlphaFold-Multimer can correctly predict IDR interactions, their dynamics, and their binding patterns with specific partners (116).

#### 3.1.2 TASSER and the I−TASSER series models

The TASSER (Threading ASSEmbly Refinement) model and its subsequently developed I−TASSER series represent a classic artificial intelligence−driven protein structure prediction framework that integrates template alignment, statistical potential functions, and computational simulation. Unlike pure template−based modeling or end−to−end deep learning approaches, the core idea of TASSER is to first map the protein sequence to potential folding template space, and then perform conformational recombination and energy optimization under a coarse−grained representation (146–148). This allows the generation of plausible three−dimensional folding models even when template information is incomplete or structurally biased. Building on this foundation, I−TASSER further introduces multi−threading alignment (LOMETS) (149) and Monte Carlo sampling strategies (150), enabling it to maintain leading performance over multiple rounds of the CASP structure prediction assessment (151–153).

In recent years, the integration of the I−TASSER framework with deep learning models has significantly expanded its structural prediction capabilities, particularly in modeling multi−domain proteins. To address the widespread presence of multi−domain proteins in biological systems, the I−TASSER−MTD pipeline incorporates deep neural network−based spatial constraint predictions (such as DeepPotential) and combines them with structure assembly algorithms like DEMO (146). This enables automated domain identification, single−domain modeling, and full−length structure assembly starting from sequence alone. This “divide− and−assemble” strategy effectively mitigates the modeling challenges posed by multi−domain proteins, which often exhibit high conformational freedom and limited structural templates.Unlike end-to-end deep learning models such as AlphaFold, the hierarchical assembly strategy of I-TASSER exhibits structural advantages in handling multi-domain proteins (125). I-TASSER employs an explicit “divide−and−assemble” strategy: starting from the amino acid sequence of the target protein, it first generates full-length atomic structural models through multiple threading alignments and iterative structural assembly simulations, followed by atomic-level structure refinement, and finally progressively infers sequence and structural feature comparisons (147). At the same time, the theoretical advantage of this modular approach lies in its effective mitigation of the high conformational entropy problem inherent to multi-domain proteins. The core challenge in multi-domain protein modeling lies in the higher degrees of freedom in domain-orientation space, which directly leads to a sharp increase in conformational entropy (154). By decomposing the high-dimensional full-length conformational search problem into multiple lower-dimensional subproblems, I-TASSER significantly reduces the difficulty of sampling the conformational space. On this basis, the assembly step reconstructs the relative orientations between domains and the spatial arrangement of flexible linkers by aligning interface residues between domains. This characteristic holds particular value for studying pathogenic proteins that contain extensive flexible linkers or exhibit aberrant inter-domain interactions (146).It is worth emphasizing that the modular framework of I-TASSER aligns well with domain-specific misfolding in neurodegenerative disease research. I-TASSER has already been used to predict the three-dimensional structure of ADO and to assess its pathological changes and consequences in neurodegenerative diseases (155); in recent years, researchers have applied I-TASSER technology to precisely study the FUS protein and to develop natural inhibitors targeting FUS protein for combating neurodegenerative diseases (156). As a result, the AI model can reconstruct reasonable full−lengthprotein structures even in the absence of complete experimental templates (151, 157, 158).

In the context of neurodegenerative disease research, the significance of the TASSER/I−TASSER system lies not only in its structural prediction accuracy but also in its systematic modeling capability for protein structure−function relationships. By integrating structure prediction with functional annotation modules (such as COFACTOR), this system can simultaneously predict molecular functions at both the domain and whole−protein levels, providing crucial insights for dissecting the potential roles of different domains of misfolded proteins in aggregation, propagation, and immune recognition (146, 159–161). Furthermore, compared to fully end−to−end deep learning models, the modularity and interpretability of I−TASSER make it more amenable to incorporating sparse experimental constraints, such as those from cryo−electron microscopy or cross−linking experiments (146, 162). This characteristic gives it a distinct advantage in studying the conformations of complex disease−associated proteins.

#### 3.1.3 RoseTTAFold

RoseTTAFold, introduced by the Baker team, features a core innovation centered on a three-track network architecture that enables the coordinated iterative updating of amino acid sequences (1D), inter-residue distance or contact information (2D), and three-dimensional coordinates (3D). This design allows the model to achieve reliable three-dimensional protein structure predictions relatively quickly and at lower computational cost, striking a favorable balance between prediction accuracy and computational efficiency (163–165).

This characteristic makes RoseTTAFold particularly well-suited for the large-scale structural modeling of misfolding-related proteins and their variants. In protein misfolding research, RoseTTAFold has been employed to predict structural frameworks and key folding motifs of disease-associated proteins such as Tau, and α-synuclein (166). These predictions provide a structural basis for analyzing the effects of mutations or abnormal modifications on protein conformational stability. While the model primarily predicts a single, stable conformation, the structural models it generates can serve as initial structures for molecular dynamics simulations and the modeling of aggregation processes, aiding in the exploration of the early stages of misfolding-to-aggregation transitions (167). Furthermore, owing to its open accessibility and computational efficiency, RoseTTAFold is widely used for the rapid expansion and refinement of protein structure databases. This, in turn, provides essential data support for subsequent investigations into the structural basis of protein misfolding and its interaction with innate immune recognition (168).

#### 3.1.4 Phyre2

Phyre2 is a template-based modeling (TBM) tool for protein structure prediction. Its core strength lies in leveraging the high conservation of protein structures during evolution. Through the use of hidden Markov models and evolutionary profile matching, it performs remote homology alignment between the target sequence and known structural databases, thereby enabling the construction of reliable three-dimensional structural models even under conditions of low sequence similarity (169, 170). This method has been widely applied in large-scale genome annotation and has demonstrated stable and reproducible predictive performance in CASP assessments (169, 171).

In misfolded protein research, Phyre2 provides important structural references for numerous disease-associated proteins that lack experimental structural data. It is particularly useful for analyzing potential structured regions and their spatial localization within proteins (169). For proteins prone to misfolding or containing low-complexity segments, such as Tau and α-synuclein, structural models predicted by Phyre2 can assist in deciphering key structural units, the spatial distribution of mutation sites, and their potential functional implications (166). This lays a structural foundation for subsequent studies on protein aggregation propensity, the formation of pathological conformations, and related immune recognition mechanisms.

#### 3.1.5 ESMFold

ESMFold, developed by Meta AI and based on the ESM-2 protein language model, performs self-supervised learning on large-scale protein sequences to capture statistical patterns and latent structural information at the sequence level. This enables three-dimensional structure prediction without requiring multiple sequence alignment (MSA). This paradigm holds significant importance in the field of protein structure prediction, offering a novel solution especially for proteins with scarce homologous sequences or insufficient evolutionary information (172–174).

In misfolded protein research, ESMFold provides a new structural prediction tool for analyzing low-homology regions, intrinsically disordered regions, and disease-associated mutations (175). Many proteins linked to neurodegenerative diseases such as Tau contain extensive intrinsically disordered regions, and their abnormal folding and aggregation are considered key factors in triggering innate immune responses (166). The structural predictions generated by ESMFold can offer preliminary structural insights into identifying potential aggregation-prone regions, immune receptor recognition sites, and protein-protein interaction interfaces (176, 177). Furthermore, its high-throughput prediction capability creates opportunities for systematically analyzing the conformational diversity of misfolded proteins and its relationship with disease progression (176).

It should be noted that ESMFold still faces challenges in prediction stability when dealing with highly complex or extreme conformational states. Therefore, its results typically need to be integrated with molecular simulations, experimental data, or other AI models to enhance the interpretability of misfolding and immune activation mechanisms (174).Building upon these foundational capabilities, the subsequent ESM-3 model has extended the paradigm from structure prediction to generative protein engineering, with recent studies demonstrating its practical utility.

Recent studies have demonstrated the practical utility of ESM-3 and ESMFold in protein function prediction and design. For clathrin identification, ClathPLM integrated embeddings from ESM-3 with CNN and attention mechanisms to achieve state-of-the-art classification performance (178). In antimicrobial peptide engineering, ESM-3 was used to generate analogs of the pseudothionin Pth-Ca1 with enhanced net charge, hydrophobicity, and helical content, while ESMFold validated their structures; the optimized peptide Design 1867 exhibited potent antibacterial activity via a barrel-stave membrane disruption mechanism (179). These examples illustrate that ESM-3 extends beyond structure prediction to generative protein engineering, offering new strategies for studying protein misfolding and its interactions with the innate immune system.

### 3.2 AI-driven dissection of innate immune responses

Innate immune responses in the central nervous system, particularly neuroinflammatory processes driven by microglia and astrocytes, are characterized by pronounced cellular heterogeneity, dynamic state transitions, and complex regulatory networks. Conventional analytical approaches are often insufficient to capture these features at single-cell and spatial resolutions. In recent years, artificial intelligence–driven analyses of single-cell and spatial multi-omics data have provided a powerful framework for dissecting immune responses in the nervous system. By enabling high-dimensional feature extraction, immune phenotype classification, and dynamic modeling, AI approaches facilitate the identification of immune cell subpopulations and activation states, the reconstruction of continuous state-transition trajectories during neuroinflammation, and the systematic modeling of neuroinflammatory regulatory networks. This section focuses on AI-based strategies for single-cell and spatial multi-omics analysis, immune phenotyping and trajectory inference, and the construction of neuroinflammatory networks, highlighting their collective contribution to understanding innate immune mechanisms in neurodegenerative diseases.

#### 3.2.1 AI−driven analysis of single−cell and spatial multi−omics

**Figure 5:**
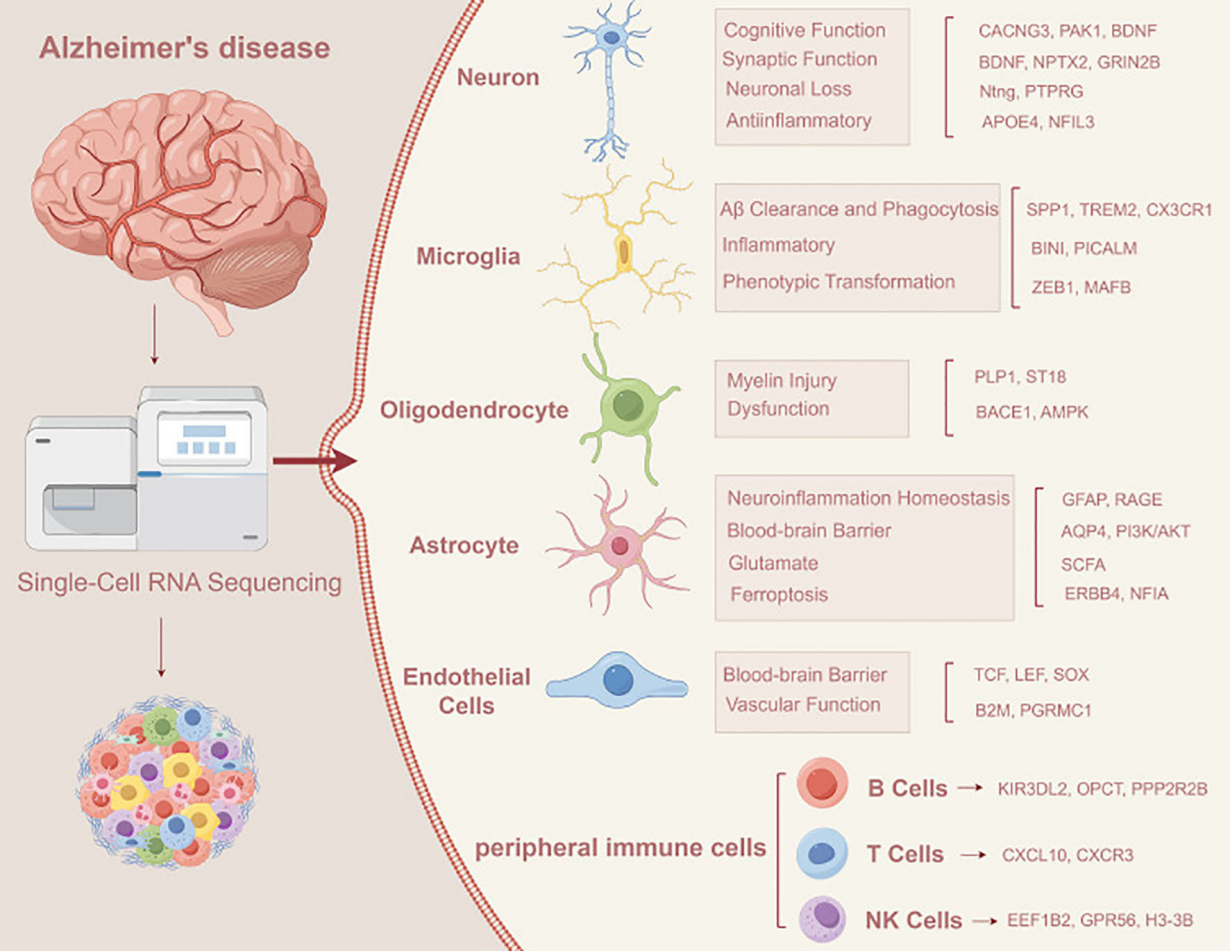
Application of scRNA-seq in Alzheimer’s disease (188). Summary of scRNA-seq applications in AD mechanistic exploration and biomarker identification: transcriptomic profiling of heterogeneous cell populations (neurons, glia, central/peripheral immune cells) in AD brain tissues and peripheral blood; screening of AD-associated cell subsets (pro-inflammatory microglia, reactive astrocytes) and molecular markers (KIR3DL2, CXCL10, EEF1B2); and construction of intercellular regulatory networks to elucidate peripheral immune cell-mediated central neuroinflammation and identify potential peripheral blood biomarkers for early AD diagnosis.

Single−cell omics technologies have revealed the high heterogeneity of various cell types and their transcriptional profiles within the nervous system, which is often obscured in traditional bulk−tissue analyses (180–184). Utilizing single−cell RNA−seq (scRNA−seq) data, researchers can identify inflammation−related cell subpopulations, such as pro−inflammatory activated microglia and reactive astrocytes, and decipher their associations with neurodegenerative progression (185–187). In studies of Alzheimer’s disease (AD) brain tissues, scRNA−seq has been employed to uncover cell states and molecular markers linked to disease progression, providing foundational data for reconstructing immune−inflammatory networks. Through in-depth analysis of specific cell types such as astrocytes and endothelial cells, research has revealed their specific roles in key physio-pathological processes including cognitive function, synaptic function, neuroinflammatory homeostasis, and blood-brain barrier maintenance. Further integration of multi−omics data through AI−driven approaches can enhance the understanding of neuroinflammatory mechanisms and improve disease−subtype classification (187, 188). For instance, transcriptomic analysis of peripheral immune cells (e.g., B cells, T cells, NK cells) can identify specific molecular markers such as KIR3DL2, CXCL10, and EEF1B2, elucidating how these cells participate in neuroinflammation and injury through distinct pathways (188) (**Figure 5**).

Summary of scRNA-seq applications in AD mechanistic exploration and biomarker identification: transcriptomic profiling of heterogeneous cell populations (neurons, glia, central/peripheral immune cells) in AD brain tissues and peripheral blood; screening of AD-associated cell subsets (pro-inflammatory microglia, reactive astrocytes) and molecular markers (KIR3DL2, CXCL10, EEF1B2); and construction of intercellular regulatory networks to elucidate peripheral immune cell-mediated central neuroinflammation and identify potential peripheral blood biomarkers for early AD diagnosis.

Spatial omics enables the simultaneous acquisition of gene expression and spatial information at tissue−site resolution, allowing the reconstruction of cellular distribution, cell−cell proximity relationships, and microenvironmental effects (189, 190). By combining AI models (e.g., CNNs (191), GNNs (192)) with spatial transcriptomic data, pattern recognition and spatial clustering can be performed to analyze the distribution and propagation patterns of inflammatory signals across different tissue regions (189, 193). This aids in identifying neuroinflammatory hotspots and characterizing their network features (194, 195).

#### 3.2.2 Immune phenotype classification and trajectory inference

In recent years, artificial intelligence has played a central role in immune phenotype classification. Deep generative models, such as variational autoencoders applied to single-cell transcriptomic data, can learn informative low-dimensional representations from high-dimensional, noisy scRNA-seq data, substantially improving downstream tasks such as clustering and cell state identification without requiring predefined marker genes. Frameworks such as scVI (Single-cell Variational Inference) leverage probabilistic modeling of gene expression to automatically distinguish immune cell subpopulations and latent states, offering a more objective and comprehensive immune phenotyping. This capability is particularly valuable for discriminating pro-inflammatory versus anti-inflammatory phenotypes and for defining activation or suppression states, forming a foundation for constructing complex disease immune networks (196).

**Figure 6:**
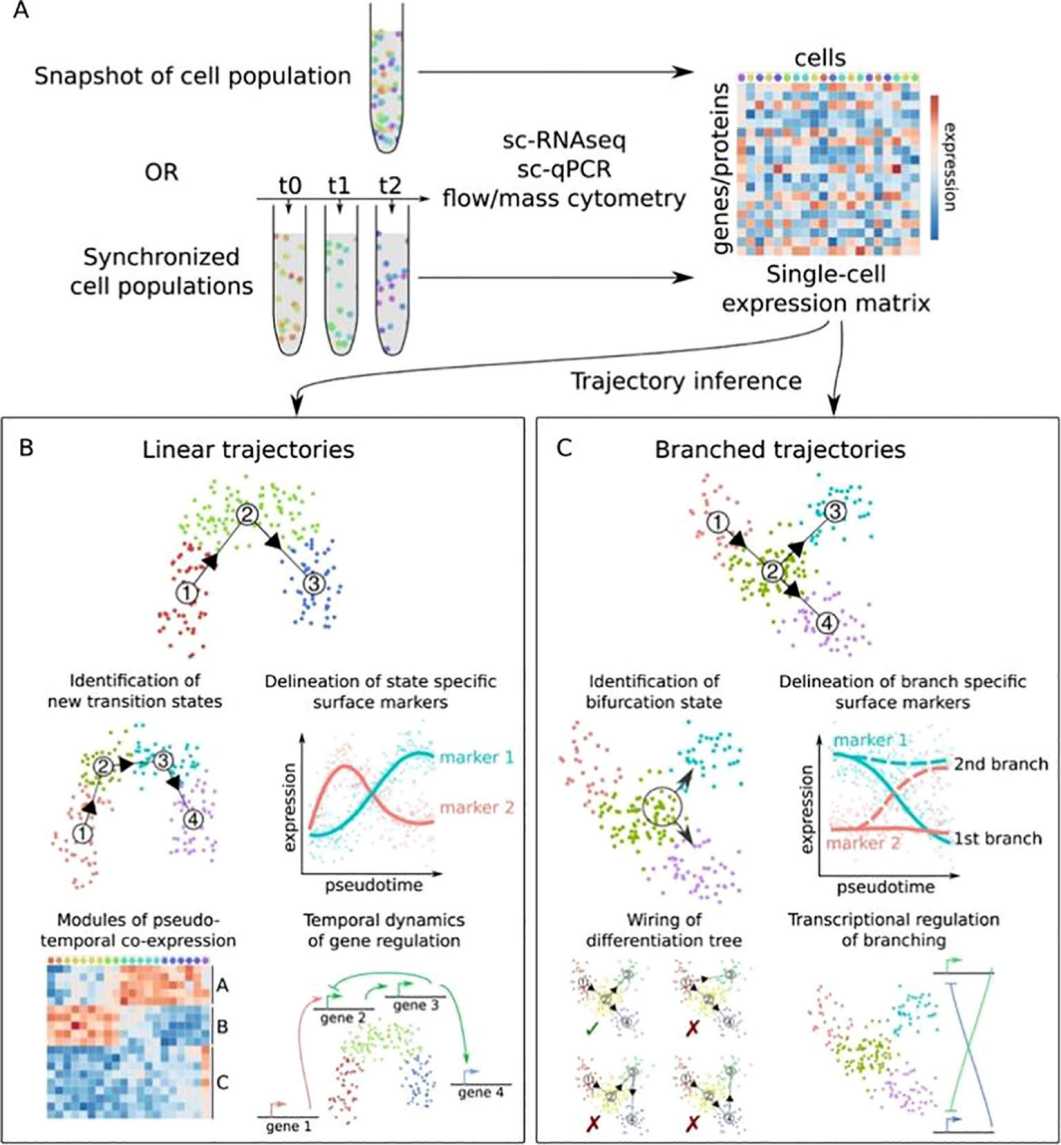
Applications of single-cell trajectory inference in neuroinflammation and innate immune responses (197). Three-panel illustration of single-cell trajectory inference applications in neuroinflammation research: **(A)** Core principle: reconstructing immune cell pseudotime developmental trajectories from static scRNA-seq data to characterize dynamic state transitions; **(B)** Microglial phenotypic dynamics: capturing intermediate transitional states during microglial shift from resting to activated phenotypes in neuroinflammation; **(C)** Branched trajectory analysis: identifying fate decision checkpoints of innate immune cells, distinguishing pro-/anti-inflammatory differentiation pathways, and reconstructing neuroinflammation-associated gene regulatory networks to mine core immune activation regulators.

Furthermore, Trajectory inference algorithms reconstruct dynamic immune cell state transitions from static single-cell data (**Figure 6A**), enabling the ordering of cells along a pseudotime axis to capture continuous activation processes such as intermediate microglial states during neuroinflammation (**Figure 6B**). Furthermore, branched trajectory models reveal bifurcation points in immune cell fate decisions, allowing the discrimination of pro- and anti-inflammatory phenotypes and the reconstruction of gene regulatory networks underlying innate immune responses in neurodegenerative diseases (**Figure 6C**) (197).

#### 3.2.3 Modeling neuroinflammatory networks

AI provides a novel methodological foundation for constructing complex inflammatory regulatory networks. By integrating multiple omics data layers, such as transcriptomics, proteomics, and metabolomics, with clinical phenotype data, AI driven network models can reveal interactions among inflammatory pathways, their regulatory relationships, and corresponding dynamic patterns of change (198, 199). A number of deep learning and graph based methods, including graph neural networks (200) and knowledge graphs (201), have been used to build immune response networks, which systematically integrate information on gene regulation, signal transduction, and cell to cell communication. These network models can not only identify key immune regulatory factors, but also predict potential points of intervention and candidate drug targets, thereby advancing a holistic understanding of the mechanisms driving inflammation (200, 201).

## 4 Integrative AI models linking protein misfolding and innate immunity

Traditional molecular biology studies often examine protein misfolding (20, 202–204)or neuroinflammation (205–207)in isolation, making it difficult to systematically reveal the dynamic, bidirectional interaction networks between the two (208). Artificial intelligence, particularly the integration of multimodal learning, causal inference, and multiscale modeling techniques (209)—which are becoming increasingly advanced alongside the rising incidence of neurodegenerative diseases (210)—can help integrate multidimensional information ranging from atomic conformations to cell populations, and from static snapshots to dynamic processes (211). This allows for the direct construction and validation of mechanistic links between “misfolding and immune sensing” (212).

### 4.1 Multimodal data fusion framework

There are many potential shared pathogenic mechanisms in neurodegenerative diseases (213). Among them, the interaction between protein misfolding and innate immunity occurs across multiple scales: specific conformations of misfolded proteins are recognized by pattern recognition receptors on the surface of microglia and astrocytes, triggering intracellular signaling cascades and altering cellular states, ultimately affecting the function of entire neural circuits (214). This process spans from the molecular and cellular scales to the network and ultimately the system scale, and is even linked to the homeostasis and metabolism of metal ions (215, 216).

An AI-driven multimodal fusion framework can align and integrate these heterogeneous data. For example, using graph neural networks (217), protein surface features predicted by AlphaFold2 or RoseTTAFold can be correlated with receptor expression profiles extracted from single-cell RNA sequencing data (218), enabling the prediction and learning of dynamic protein behaviors, conformational changes, and protein-protein interactions (174). For instance, SAGEFusionNet can assist supervised graph neural networks in predicting brain age as a biomarker for neurodegeneration (219). The GASIDN model utilizes a 1D convolutional module on protein sequences and a graph learning module built from AlphaFold2-derived contact maps to extract multidimensional features (220). Going further, by combining spatial transcriptomics data (221), AI models can reveal the key role of NLRP3 in the pathogenesis of neurological disorders (222), elucidate Toll-like receptor-mediated neuroinflammation in neurodegenerative diseases (223), and locate immune cells expressing specific PRRs in the spatial distribution patterns around Aβ plaques (224). This helps uncover which protein conformations are most immunogenic in which spatial contexts. This multimodal mapping of “structure-expression-space” is key to understanding how misfolded proteins act as DAMPs and are specifically recognized (225).

### 4.2 AI-based prediction, diagnosis, and therapeutic applications for neurodegenerative disease pathology

AI is a broad term representing new technologies that use machines and computer systems to simulate and extend human intelligence. It includes methods such as ML, DL, and NLP. In the era of big data, AI has become a crucial tool for enhancing the detection of neurodegenerative diseases (226, 227). The combination of AI with mathematical and physical models is now being used to simulate the dynamic aggregation and intercellular propagation of misfolded proteins. For example, GANs (228) and Diffusion models (229, 230) can learn the structural features of known pathological aggregates to generate a vast number of possible intermediate or variant conformations. These are used to predict their tendency to interact with cell membranes and be endocytosed, and to develop therapeutics targeting key regulators of neuroinflammation such as Gal-3 (231–233).

.Regarding the diagnosis and treatment of neurodegenerative diseases, the cGAN-based architecture model ST-cGAN is used for cross-modality synthesis of MR images and applied to the detection and diagnosis of neurodegenerative diseases (234). Meanwhile, the combination of biomarkers with AI has become a key tool in personalized medicine for neurodegenerative diseases (235). However, as many biomarkers remain confined to preclinical research and face translational challenges due to species-specific differences, lack of standardization, and clinical heterogeneity, AI-driven multimodal data integration offers new opportunities. It aligns biomarker profiles with evolving disease states and improves patient stratification based on the current landscape (236).

### 4.3 A cross-disease, cross-scale unified modeling framework

Although different types of neurodegenerative diseases involve distinct core pathological proteins, they share commonalities along the “misfolding-neuroinflammation” axis mechanism (237, 238). The use of AI can facilitate the construction of unified frameworks for disease prediction based on single-cell omics (239), or even cross-disease unified computational frameworks. Beyond integrating and modeling large-scale data within the same modality, cross-disease knowledge transfer is another key strategy to address data scarcity in rare neurodegenerative diseases (240, 241). For instance, the disease knowledge transfer framework proposed by Marinescu et al., which operates under the hypothesis of shared biomarker kinetics across different diseases, successfully transferred multimodal information from typical Alzheimer’s disease to the study of posterior cortical atrophy (242). The use of multimodal models, such as deep learning-based approaches, for predicting brain age, cognition, and amyloid pathology (243) provides an important methodological extension for our AI diagnostic systems based on proteomics. Furthermore, models built on structural magnetic resonance imaging (sMRI) and blood parameters currently show good potential for brain health assessment (244). Researchers can simulate the global trajectories of protein misfolding and immune system interactions under different genetic backgrounds and environmental factors *in silico*, thereby identifying common vulnerable nodes and personalized intervention strategies.

## 5 Challenges, future perspectives, and conclusions

In recent years, brain science has undoubtedly entered a new era, thanks to a large number of methodological advances and the digital realization of data integration and modeling, spanning from molecules to the whole brain. Significant progress is emerging at the intersection of neuroscience, technology, and computation (245). Although AI brings revolutionary tools for deciphering the complex relationship between protein misfolding and innate immunity in neurodegenerative diseases, its development and application still face multiple challenges, while also pointing to new directions for future research.

The developmental prospects for artificial intelligence in the field of neurodegenerative diseases lie at its core evolution from an assistive tool to a trustworthy clinical partner. The future will focus on enhancing the accuracy of early diagnosis and the establishment of clinician trust by integrating high-quality multimodal data and developing interpretable decision models. Through deep integration with electronic medical record systems, a leap from static analysis to dynamic disease course management can be achieved, providing patients with personalized risk prediction and intervention plans. Ultimately, AI will propel the diagnosis and treatment of neurological diseases towards a new stage characterized by greater precision, efficiency, and accessibility.

The performance of current AI models heavily depends on the quality and breadth of training data. As neuroscience research continues to evolve, experimental datasets are becoming increasingly large and complex. Advanced data science tools play a central role in neuroscience research (246), but conventional AI often fails to capture the complex underlying distributions of multimodal health data and the long-term dependencies throughout medical histories (247). Furthermore, neuroscience research data commonly suffers from limited sample sizes, and the research landscape still exhibits significant ‘fragmentation’ (248). The immune environment of the central nervous system is highly specialized, characterized by the presence of the blood-brain barrier, the unique developmental origin and dynamic surveillance functions of microglia, and the tight interactions between neurons and glial cells (249, 250). GAI models used in the diagnosis of neurodegenerative diseases, such as Alzheimer’s and Parkinson’s disease, often face issues affecting their generalizability, such as insufficient training data or lack of sample representativeness, can generate inaccurate or entirely fabricated medical information, and pose risks of data leakage and misuse (251).

Despite the substantial potential of artificial intelligence in the field of neurodegenerative diseases as demonstrated by the aforementioned studies, current AI models still harbor several inherent limitations that warrant systematic scrutiny. First, most deep learning-based structure prediction models, including AlphaFold and ESMFold, are designed to predict a single thermodynamically stable conformation (252, 253). This intrinsic characteristic renders them inherently inadequate for capturing conformational heterogeneity, kinetic intermediates, and misfolding trajectories, which are precisely the core pathological features of neurodegenerative diseases. Although low-confidence (pLDDT) regions predicted by these models offer suggestive insights, they cannot be directly equated with the dynamic ensemble of pathological conformations (133). Second, model performance is highly dependent on the quality, diversity, and evolutionary depth of the training data; for proteins with scarce homologous sequences or those containing extensive intrinsically disordered regions, existing models still face formidable challenges. Third, although multimodal and generative models offer new possibilities for exploring conformational space, their outputs still necessitate rigorous experimental validation. Finally, the translation of AI-driven research findings into clinical applications is hampered by several obstacles, including insufficient model interpretability, limited generalizability across diverse patient populations, and difficulties in integration with existing clinical workflows. Overcoming these limitations urgently calls for deep collaboration among computational scientists, structural biologists, and clinical researchers to collectively develop a new generation of AI models that integrate predictive accuracy with biological relevance and clinical applicability.

Neurodegenerative diseases arise from a tightly coupled pathogenic axis involving protein misfolding, abnormal aggregation, and persistent innate immune activation. Misfolded proteins such as amyloid-β, Tau, α-synuclein, and TDP-43 act not only as neurotoxic species but also as damage-associated molecular patterns that engage pattern recognition receptors on microglia and astrocytes, driving chronic neuroinflammation and disease progression. This review highlights how artificial intelligence is reshaping the investigation of this misfolding–immunity interplay across multiple scales. AI-based protein structure prediction tools provide critical structural insights into disease-associated proteins, while deep learning approaches applied to single-cell and spatial multi-omics data enable high-resolution mapping of innate immune heterogeneity and inflammatory trajectories in the central nervous system. Emerging multimodal and graph-based frameworks further integrate protein conformational features with immune signaling networks, offering mechanistic links between specific misfolded states and immune sensing. This review centers on the application of artificial intelligence to accelerate progress in the diagnosis and treatment of neurodegenerative diseases. Although the pathological mechanisms underlying pediatric forms differ from those of adult-onset disorders and pose greater diagnostic challenges (254), AI holds significant promise as a transformative approach to overcoming these obstacles. Nevertheless, further research is urgently needed to fully realize this potential in the context of childhood neurodegeneration. Although challenges related to data quality, model interpretability, and clinical translation remain, AI-driven integrative modeling is poised to transform neurodegenerative disease research by enabling system-level understanding, improving disease stratification, and accelerating the development of precise diagnostic and therapeutic strategies.
