# Correction: AI-driven insights into protein misfolding and innate immunity in neurodegenerative diseases

**DOI:** 10.3389/fimmu.2026.1879874

**Published:** 2026-07-14

**Authors:** Hui Xin Deng, Jing Ling Cao, Yao Wu, Si Jin Jiang, Qian Qian Fang, Bi Yue Zhu, Yong Jian Jiang

**Affiliations:** Department of Pharmacy Children’s Hospital of Chongqing Medical University, National Clinical Research Center for Children and Adolescents’Health and Diseases, Ministry of Education Key Laboratory of Child Development and Disorders, International Science and Technology Cooperation Base of Child Development and Critical Disorders, Chongqing Key Laboratory of Child, Neurodevelopment and Cognitive Disorders, Intelligent Application of Big Data in Pediatrics Engineering Research Center of Chongqing Education Commission of China, Chongqing, China

**Keywords:** artificial intelligence, innate immune, misfolded proteins, neurodegenerative disease, neuroinflammation

There was a mistake in **Figure 3** as published. **Figure 3** was replaced due to unresolved permission rights for the original figure. The new figure has full permission clearance. The corrected **Figure 3** appears below.

**Figure 3:**
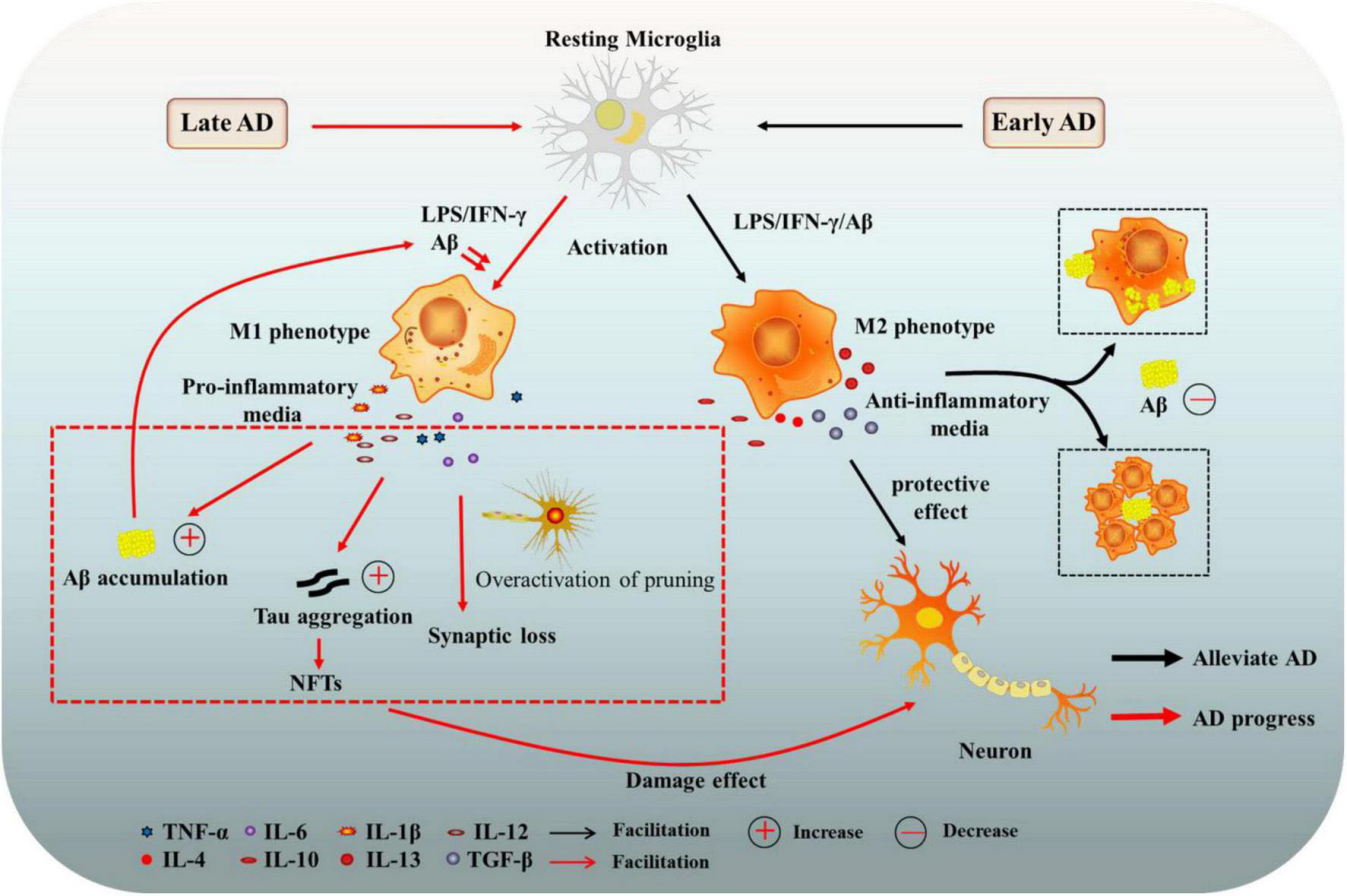
The role of microglia in Alzheimer’s disease (AD). Microglia can have beneficial or harmful effects on AD. In the early stage of AD, the activation state of microglia is mainly a protective phenotype (M2), secreting anti-inflammatory mediators and neurotrophic factors, and clearing or isolating Aβ and Tau proteins, contributing to the protection of neurons. In late AD, microglia tend to be activated as a harmful phenotype (M1), enhancing the release of pro-inflammatory factors, which damage neurons and promote the progression of AD. AD, Alzheimer’s disease; Aβ, amyloid beta-protein; NFTs, neurofibrillary tangles; LPS, lipopolysaccharide; TGF-β, transforming growth factor-β; IFN-γ, interferon γ; TNF-α, tumor necrosis factor-α; IL-6, interleukin-6.

There was a mistake in the caption of **Figure 3** as published. The figure caption was displayed as “Microglia regulate neuronal networks through engulfing apoptotic neurons via TAM receptor-mediated recognition of GAS6-opsonized cells, modulating synaptic plasticity by secreting ROS to downregulate AMPA receptors and BDNF to engage Trk receptors, stripping synapses tagged by complement and through CX3CR1-CX3CL1 interactions, and indirectly influencing neuronal activity by releasing TNF-athat stimulates astrocytes to release glutamate and ATP”. The corrected caption of **Figure 3** appears below.

The role of microglia in Alzheimer’s disease (AD). Microglia can have beneficial or harmful effects on AD. In the early stage of AD, the activation state of microglia is mainly a protective phenotype (M2), secreting anti-inflammatory mediators and neurotrophic factors, and clearing or isolating Ab and Tau proteins, contributing to the protection of neurons. In late AD, microglia tend to be activated as a harmful phenotype (M1), enhancing the release of pro-inflammatory factors, which damage neurons and promote the progression of AD. AD, Alzheimer’s disease; Ab, amyloid beta-protein; NFTs, neurofibrillary tangles; LPS, lipopolysaccharide; TGF-b, transforming growth factor-b; IFN-g, interferon g; TNF-a, tumor necrosis factor-a; IL-6, interleukin-6.

The original version of this article has been updated.

